# B-cell receptor profiling before and after IVIG monotherapy in newly diagnosed idiopathic inflammatory myopathies

**DOI:** 10.1093/rheumatology/keac602

**Published:** 2022-11-02

**Authors:** Dornatien C Anang, Hannah A W Walter, Johan Lim, Ilse Niewold, Linda van der Weele, Eleonora Aronica, Filip Eftimov, Joost Raaphorst, Barbera D C van Schaik, Antoine H C van Kampen, Anneke J van der Kooi, Niek de Vries

**Affiliations:** Department of Rheumatology and Clinical Immunology, Amsterdam Rheumatology and Immunology Center, Amsterdam UMC, University of Amsterdam, Amsterdam, The Netherlands; Department of Experimental Immunology, Amsterdam Infection and Immunity Institute, Amsterdam UMC, University of Amsterdam, Amsterdam, The Netherlands; Department of Genome analysis, Amsterdam UMC, University of Amsterdam, Amsterdam, Netherlands; Department of Neurology, Amsterdam UMC, University of Amsterdam, Amsterdam Neuroscience, Amsterdam, The Netherlands; Department of Neurology, Amsterdam UMC, University of Amsterdam, Amsterdam Neuroscience, Amsterdam, The Netherlands; Department of Rheumatology and Clinical Immunology, Amsterdam Rheumatology and Immunology Center, Amsterdam UMC, University of Amsterdam, Amsterdam, The Netherlands; Department of Genome analysis, Amsterdam UMC, University of Amsterdam, Amsterdam, Netherlands; Department of Rheumatology and Clinical Immunology, Amsterdam Rheumatology and Immunology Center, Amsterdam UMC, University of Amsterdam, Amsterdam, The Netherlands; Department of Experimental Immunology, Amsterdam Infection and Immunity Institute, Amsterdam UMC, University of Amsterdam, Amsterdam, The Netherlands; Department of (Neuro)Pathology, Amsterdam Neuroscience, Amsterdam UMC, University of Amsterdam, Amsterdam, The Netherlands; Department of Neurology, Amsterdam UMC, University of Amsterdam, Amsterdam Neuroscience, Amsterdam, The Netherlands; Department of Neurology, Amsterdam UMC, University of Amsterdam, Amsterdam Neuroscience, Amsterdam, The Netherlands; Bioinformatics Laboratory, Department of Epidemiology and Data science, Amsterdam Public Health Institute, Amsterdam Infection and Immunity Institute, Amsterdam UMC, University of Amsterdam, Amsterdam, The Netherlands; Bioinformatics Laboratory, Department of Epidemiology and Data science, Amsterdam Public Health Institute, Amsterdam Infection and Immunity Institute, Amsterdam UMC, University of Amsterdam, Amsterdam, The Netherlands; Department of Neurology, Amsterdam UMC, University of Amsterdam, Amsterdam Neuroscience, Amsterdam, The Netherlands; Department of Rheumatology and Clinical Immunology, Amsterdam Rheumatology and Immunology Center, Amsterdam UMC, University of Amsterdam, Amsterdam, The Netherlands; Department of Experimental Immunology, Amsterdam Infection and Immunity Institute, Amsterdam UMC, University of Amsterdam, Amsterdam, The Netherlands

**Keywords:** Idiopathic inflammatory myopathy, B-cell receptor sequencing, B cells, intravenous immunoglobulin

## Abstract

**Objective:**

To unravel B-cell receptor (BcR) characteristics in muscle tissues and peripheral blood and gain more insight into BcR repertoire changes in peripheral blood in idiopathic inflammatory myopathies (IIMs), and study how this correlates to the clinical response to IVIG.

**Methods:**

Nineteen treatment-naive patients with newly diagnosed IIM were prospectively treated with IVIG monotherapy. RNA-based BcR repertoire sequencing was performed in muscle biopsies collected before, and in peripheral blood (PB) collected before and nine weeks after IVIG treatment. Results were correlated to patients’ clinical improvement based on the total improvement score (TIS).

**Results:**

Prior to IVIG treatment, BcR clones found in muscle tissue could be retrieved in peripheral blood. Nine weeks after IVIG treatment, new patient-specific dominant BcR clones appeared in peripheral blood while pre-treatment dominant BcR clones disappeared. The cumulative frequency of all dominant BcR clones before treatment was significantly higher in individuals who responded to IVIG compared with those who did not respond to IVIG, and correlated with a higher CK. During follow-up, a decrease in the cumulative frequency of all dominant clones correlated with a higher TIS.

**Conclusion:**

In treatment-naive patients with newly diagnosed IIM, muscle tissue and peripheral blood share expanded BcR clones. In our study a higher cumulative frequency of dominant BcR clones in blood before treatment was associated with a higher CK and better treatment response, suggesting that response to IVIG may depend on the composition of the pre-treatment BcR repertoire.

Rheumatology key messages

- In IIM patients (dominant) BcR clones in muscle tissues can be retrieved in peripheral blood.
- New patient-specific dominant clones are formed and pre-treatment dominant clones disappear nine weeks after IVIG treatment.
- IIM patients with a high impact of dominant clones in blood before therapy respond better to IVIG.

## Introduction

Idiopathic inflammatory myopathies (IIM), often referred to as ‘myositis’, are a heterogeneous group of auto-immune disorders characterized by subacute, often severe progressive proximal muscle weakness. Several treatable subtypes can be distinguished: i.e. DM, antisynthetase syndrome (ASS), immune-mediated necrotizing myopathy (IMNM), non-specific/overlap myositis (NM/OM) and PM [1, 2]. Polymyositis is a contested entity, which is currently considered a diagnosis after exclusion of all other forms of myositis. Here, first-line treatment consists of high dose corticosteroids, but in more than half of the patients, long-term treatment with additional immunosuppressive therapy is needed [3, 4]. However, despite combined medical treatment, the majority of patients (70%) have a chronic or polyphasic disease course and develop significant residual disability and reduced quality of life [5]. Consequently, there is a clear need for a better understanding of the disease pathogenesis, for better treatment strategies and a better understanding of their mechanism of action.

In recent years, pooled human IgG purified from healthy donors [6], IVIG, are increasingly used in the treatment of IIMs [7–9]. IVIG has been reported to bind and neutralize autoantibodies, pro-inflammatory cytokines such as TNF-alpha [10], and the B-cell activation factor (BAFF) [11–13]. Furthermore, IVIG was shown to increase the number of B cells and plasmablasts in peripheral blood of Guillain Barré syndrome (GBS) patients, and can directly affect the B-cell repertoire [14–16]. This is of interest because B cells may play a pivotal role in IIMs [17, 18]: muscle biopsies of myositis patients show plasma cells [19–21], 60% to 70% of IIM patients are positive for myositis-specific and myositis-associated antibodies [22], and B-cell directed therapies such as rituximab are effective in the treatment of myositis [23–26].

Although B cells may play a key role in IIMs, and IVIG can influence the B-cell repertoire, little information is available on modulation by IVIG of the B-cell response at the clonal level in IIM and other auto-immune diseases, and whether differences in the effect of this modulation explain the large inter-individual variability in clinical response to IVIG [27–29]. To fill this gap we used RNA-based next generation sequencing to study the B-cell receptor (BcR) repertoire in baseline muscle tissue and in peripheral blood before and 9 weeks after IVIG treatment in treatment-naive myositis patients. The results presented here provide new insights into the possible role of B cells in the disease pathogenesis and the mechanism of action of IVIG in peripheral blood over time.

## Patients and methods

### Ethical statement

All patients signed informed consent prior to inclusion in the study. This study was conducted with approval of the local medical research ethics committee of the Academic Medical Centre, Amsterdam, and in accordance with the declaration of Helsinki.

### Patients and study design

Adult, treatment-naive patients with newly diagnosed, biopsy-proven IIM were included in a 9 week phase-2 open-label study that investigated IVIG as first-line treatment in IIM patients [27]. IVIG monotherapy was started with 2 g/kg at baseline, followed by two subsequent doses of 1 g/kg at week three and week six. Patients received no glucocorticoids or other immunosuppressive treatment.

### Clinical assessment

Clinical and laboratory examination was performed at two time points: at baseline before treatment, and at follow-up after treatment at 9 weeks, or earlier in case of preliminary ending of protocol. Clinical examination consisted of all core set measures (CSM) of the IMACS (Supplementary Materials and Methods, available at *Rheumatology* online), and results in a Total Improvement Score (TIS). TIS ≥40 was defined as response to treatment [30].

### Collection and processing of muscle tissues and blood samples

Muscle biopsies were taken upon diagnosis before treatment (baseline) in all patients. For collection of muscle biopsies, the optimal biopsy location was based on the presence of oedema on muscle imaging (MRI or ultrasound). If no oedema was present, the biopsy was taken from a clinically weak muscle. Biopsies were taken according to recommended standards for muscle biopsies [31]. Muscle biopsies were stored at –80°C until RNA isolation. Peripheral blood was drawn from all patients at two time points: prior to treatment (baseline) and at week 9, after three cycles of IVIG treatment, or earlier in case of preliminary ending of the protocol (follow-up) in PAXgene blood RNA tubes (PreAnalytiX, Breda, The Netherlands). Blood samples were all stored at –80°C until RNA isolation.

### RNA isolation and next generation sequencing of the BcR repertoire

RNA isolation from peripheral blood was carried out using PAXgene blood miRNA isolation kit (Qiagen, Hilden, Germany). RNA was isolated from stored muscle biopsies after homogenization with the MagNA Lyser (Roche) as extensively described in the Supplementary Materials and Methods, available at *Rheumatology* online. Amplification of the BcR was carried out as previously described [32, 33] and further reported in the online Supplementary Materials and Methods, available at *Rheumatology* online.

### Bioinformatics generation and analysis of BcR repertoires

The obtained sequencing reads were analysed with an in-house developed pipeline for repertoire analysis ‘RESEDA’ (REpertoire SEquencing Data Analysis, https://bitbucket.org/barbera/reseda) which has been previously described extensively [34] and is further described in the online Supplementary Materials and Methods, available at *Rheumatology* online. Clones with a frequency ≥0.5% of the total repertoire were labelled dominant or highly expanded clones (HECs) [33, 34].

### Clustering related clones

Clones were grouped on the CDR3 amino acid sequence using in-house developed R scripts. In brief, the Hamming distance between clones was calculated. An approach similar to [35] was taken to calculate a dynamic threshold for grouping clones together as described in the Supplementary Materials and Methods, available at *Rheumatology* online.

### Statistical analysis

After testing for normality with D’Agostino and Pearson omnibus test, data are presented as mean (s.d.) for normally distributed data or median with IQR and analysed using the Kruskal–Wallis or Mann–Whitney test for non-normally distributed data. Correlations were calculated with Spearman's rank correlation coefficient. All statistical analyses were performed using GraphPad Prism Software (version 8.0, GraphPad Software, Inc., La Jolla, CA, USA). *P*-values <0.05 were considered statistically significant.

## Results

### Patients

Twenty newly diagnosed IIM patients participated in the IMMEDIATE study, and outcomes have been reported previously [27]. One patient was excluded earlier from analysis [1] due to inability to reach the threshold of improvement on the TIS due to a ceiling effect, thus leaving 19 patients for analysis. Seven (37%) patients ended the protocol preliminary, after a median of 6 weeks, last observation carried forward, as reported in the IMMEDIATE [27]. Eight (42%) patients had DM, five (26%) had NM/OM, five (26%) had an IMNM and one patient (5%) had ASS. All patients had proximal muscle weakness at inclusion. Muscle biopsies were taken from the vastus lateralis muscle in 16 patients, from the triceps in two patients and from the deltoid in one patient. All biopsied muscles showed oedema on magnetic resonance imaging T2-weighed Dixon scans. Patient characteristics are summarized in Table 1 and Supplementary Table S2 and S3, available at *Rheumatology* online.

**Table 1.** Baseline characteristics of the 19 included patients

Characteristic	Patients (n = 19)
Age at onset in years, median (IQR)	59 (37–69)
Months between start of symptoms until diagnosis, median (IQR)	5 (3–6)
Gender, females, *n* (%)	12 (63)
Connective tissue disorder, *n* (%)^a^	3 (16)
Cancer, *n* (%)	1 (5)
Serum CK, median (IQR), U/L	1199 (179–6500)
Myositis-specific and myositis associated antibodies, *n* (%)	
Anti-HMGCR	3 (16)
Anti-NXP2	3 (16)
Anti-Jo1	1 (5)
Anti-MDA5	1 (5)
Anti-SRP	1 (5)
Anti-TIF1gamma	1 (5)
Myositis associated antibodies (MAA) only	2 (11)
Seronegative	7 (37)
Responders, TIS ≥40 at 9 weeks, *n* (%)	8 (42)

a *n* = 1 MCTD, *n* = 1 SS, *n* = 1 SSc.

CK: creatine kinase; IQR: interquartile range; U/L: units per litre.

### Dominant BcR clones are present in muscle tissue and peripheral blood of myositis patients prior to IVIG treatment

**Figure 1.**
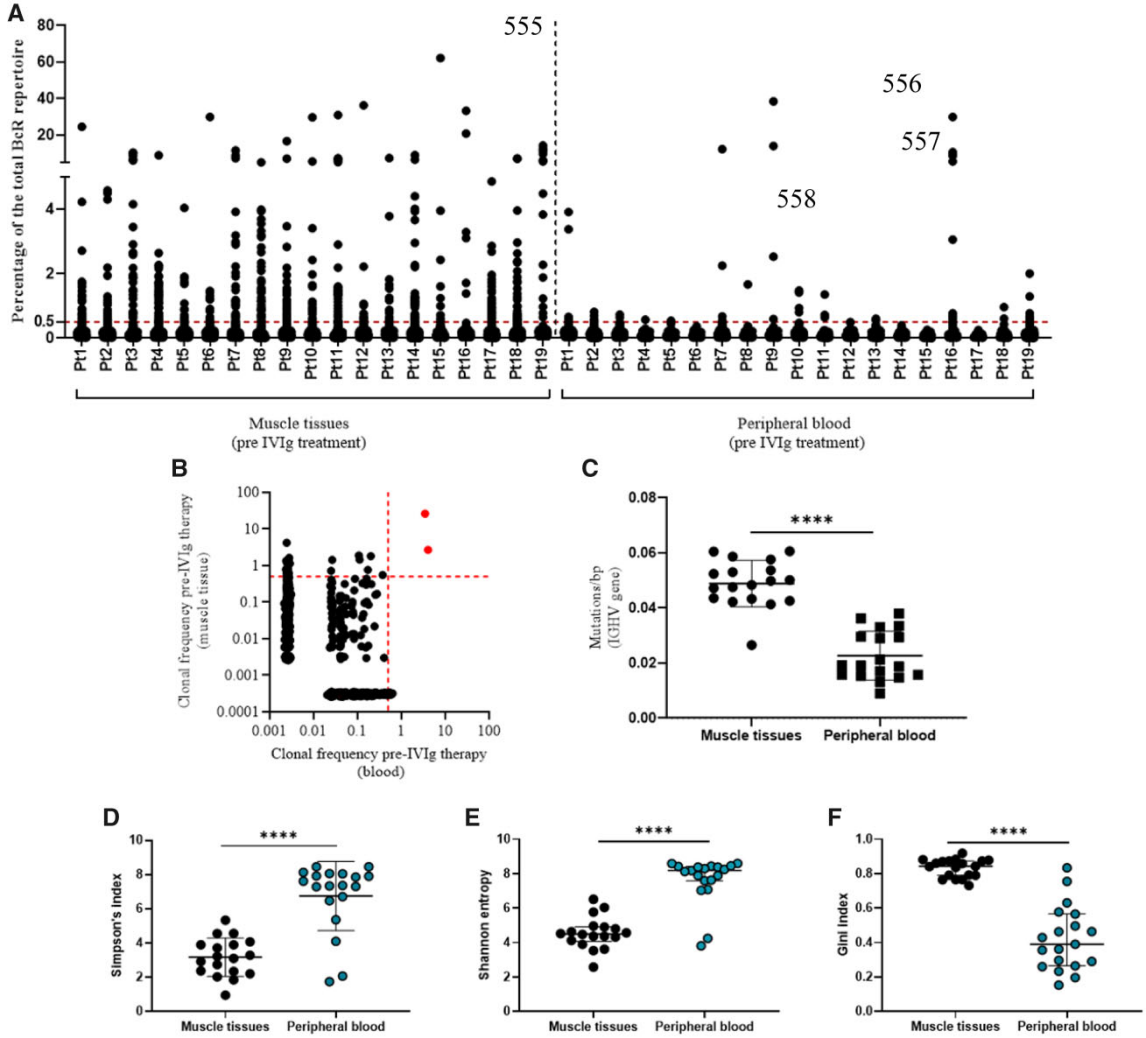
Identification of BcR clones in muscle tissues and peripheral blood before IVIG treatment. **(A)** Scatter plot of the BcR repertoire in muscle tissues and peripheral blood of IIM patients prior to IVIG treatment. Each dot represents a unique BcR clone. **(B)** Representative example of CDR3 clonal overlap between peripheral blood and muscle tissue pre-IVIG treatment, shown for patient one (Pt1). Each dot represents a unique BcR clone, and its frequency in the analysed repertoire is depicted on the x (blood pre-IVIG treatment) and y (muscle tissue pre-IVIG treatment) axes as percentage of total UMIs. The dotted lines on each axis indicate the 0.5% cut-off for dominant BcR clones. BcR clones in red are clones which are dominant in both samples. **(C)** Mutation load [expressed as mutations/base pair (bp)] of BcR clones detected in muscle tissues and peripheral blood prior to IVIG treatment. (**D**–**F**) Simpson’s index, Shannon entropy and Gini index to evaluate the diversity of the BcR repertoires in peripheral blood and muscle tissues at baseline

To get a better understanding of B-cell involvement in IIMs, we started by investigating the hypothesis that in IIMs, muscle biopsies and peripheral blood harbour expanded BcR clones. We sequenced the BcR heavy chain repertoires in muscle tissue and peripheral blood obtained in all patients prior to IVIG treatment and detected multiple dominant BcR clones in all 19 muscle tissues sequenced and in 14 of the 19 peripheral blood samples sequenced (Fig. 1A). Since (dominant) BcR clones were detected in both muscle tissue and peripheral blood prior to IVIG treatment, we next investigated whether the (dominant) BcR clones present in muscle tissues were the same (dominant) BcR clones present in peripheral blood. In 18 of the 19 patients, BcR clones found in muscle tissue could be detected in peripheral blood. In three of these patients, BcR clones which were dominant (clones with a frequency ≥0.5% of the total BcR repertoire) in muscle tissues were also dominant in peripheral blood of those patients (Fig. 1B and Supplementary Table S1, available at *Rheumatology* online). We next investigated whether BcR clones found in both muscle tissues and peripheral blood shared similar characteristics. From each patient, we performed a clustering analysis at the CDR3 level of the top 100 most expanded clones shared between muscle tissues and peripheral blood. Indeed, some of these BcR clones in each patient clustered together based on CDR3 amino acid similarity (Supplementary Fig. S1A, available at *Rheumatology* online). Additional analysis on these shared clones showed a preferential usage of certain V-J gene combinations with IGHV4-31, IGHV3-23, IGHV3-3 and IGHV3-74 the most used V genes in muscle tissues, IGHV1-2 and IGHV3-3 the most used V genes in blood and JH4 the most used J gene segment in both blood and muscle tissue (Supplementary Fig. S1B and 1C, available at *Rheumatology* online). We next investigated whether the different myositis subtypes showed different BcR clonal repertoires (the number and impact of dominant BcR clones) as well as other BcR repertoire features such as CDR3 length, CDR3 charge and VJ gene usage. Subgroup analysis based on antibody type [myositis specific antibodies (MSA), myositis associated antibodies (MAA) and seronegative] (Supplementary Fig. S2, available at *Rheumatology* online) or myositis subtype, even in the largest group of eight DM patients, did not show significant differences (data not shown). The total number of clones retrieved from each antibody subgroup is further listed in Supplementary Table S2, available at *Rheumatology* online.

To further explore the phenotypic composition of the BcR repertoire in muscle tissues and peripheral blood prior to IVIG treatment, we analysed the mutation load of the BcR heavy chain variable region (IGHV) as an indication of the maturation status of the BcR repertoire. A high mutation load indicates that the repertoire is dominated by mature BcR clones (i.e. memory and plasma blasts/cells), while a low mutation load indicates that the BcR repertoire is mainly composed of immature BcR clones (naive). When we compared the mutation load of the BcR repertoire obtained from muscle tissues to that of peripheral blood, the BcR repertoire in muscle tissues had a significantly higher mutation load compared with the pretreatment BcR repertoire obtained from peripheral blood (*P* < 0.0001) (Fig. 1C). Finally, we compared the diversities of the BcR repertoires obtained from peripheral blood and muscle tissues at baseline using the Simpson’s index, Shannon entropy and the Gini index (these indices are further explained in the Supplementary Materials and Methods, available at *Rheumatology* online). We observed that the peripheral blood repertoires were more diverse when compared with the repertoires in muscle tissues (Fig. 1D–F).

Taken together, our analyses in this limited number of patients show that muscle tissue and peripheral blood of IIM patients harbour dominant BcR clones. In addition, the BcR repertoire of muscle tissues is mostly composed of highly mutated BcR clones when compared with peripheral blood.

## The dynamics of BCR clonality in peripheral blood after IVIG treatment

**Figure 2.**
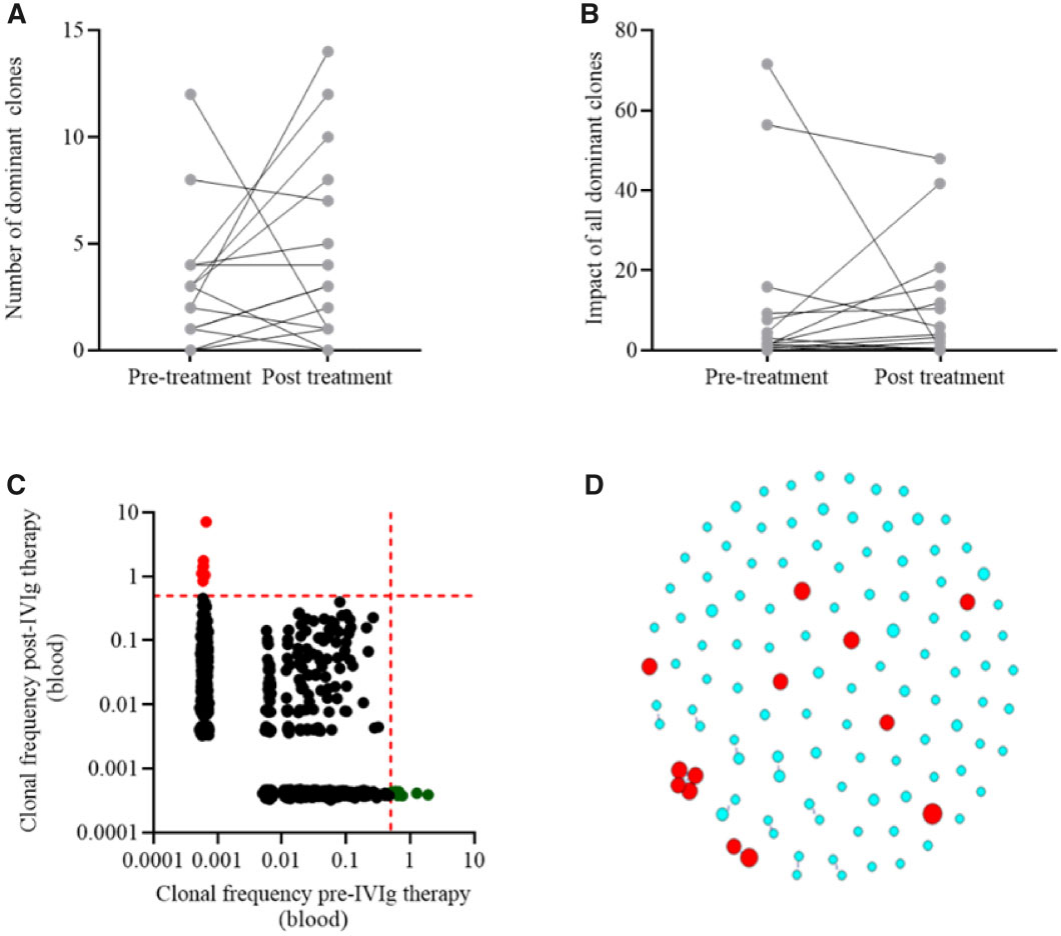
BcR repertoire characteristics in peripheral blood before and after IVIG treatment. For each patient, panel **(A)** shows the number of dominant BcR clones while panel **(B)** shows the combined impact of all dominant BcR clones on the BcR repertoire before and 9 weeks after IVIG treatment. **(C)** Representative CDR3 overlap plot before (x-axis, dominant clones in green) and after (y-axis, dominant clones in red) IVIG treatment for one patient. Each dot represents a unique BcR clone, its frequency being calculated as percentage of total UMI-corrected reads. The dotted red lines on each axis indicate the 0.5% cut-off for dominant BcR clones. **(D)** A representative example of a CDR3 amino acid cluster analysis in one patient is shown. In red are newly formed dominant BcR clones 9 weeks after IVIG treatment. Clones present at baseline (in blue) show no clustering with the newly formed red clones. For clarity, only the top 100 clones at baseline are shown

To explore the effects of IVIG treatment on the BcR repertoire, we compared the number and cumulative frequency (impact) of dominant BcR clones in peripheral blood before and after 9 weeks of IVIG treatment. At the time points, the number of dominant BcR clones (Fig. 2A) and the impact of dominant BcR clones (Fig. 2B) did not differ. After subgrouping based on the type of antibody, this analysis also did not show significant differences (Supplementary Fig. S2A–F, available at *Rheumatology* online). BcR repertoire features like CDR3 length, CDR3 charge, V and J gene usage and repertoire diversity did not differ between both time points (Supplementary Fig. S3A–C and S3F–H, available at *Rheumatology* online). However, comparing dominant clones at baseline with those present 9 weeks after IVIG treatment we observed that only one patient had a shared dominant clone between both time points (Supplementary Fig. S3D, available at *Rheumatology* online). In all other patients, dominant BcR clones present 9 weeks after treatment were completely different from the dominant clones present before treatment (Fig. 2C). Additionally, CDR3 amino acid overlap plots between different individuals showed that these newly formed dominant clones at week 9 were patient-specific as no two patients shared dominant clones both before and after IVIG treatment (Supplementary Fig. S3E, available at *Rheumatology* online).

We further explored whether the dominant clones at week 9 were *de novo* or were in any way related to clones present prior to IVIG treatment. From each patient, the CDR3 amino acid sequences of all dominant clones retrieved at week 9 were compared with the CDR3 amino acid sequences of all clones present at baseline in a clustering analysis. Our analysis revealed that dominant BcR clones at follow-up were completely different from the BcR clones pre-treatment, as no clusters were observed between clones present prior to IVIG treatment and the newly formed dominant BcR clones after IVIG (Fig. 2D).

### Patients with a high impact of dominant BcR clones in peripheral blood at baseline respond better to IVIG treatment

**Figure 3.**
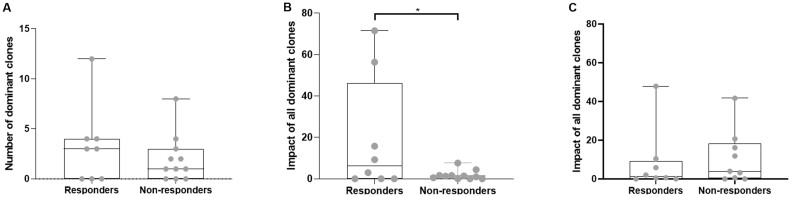
BcR repertoire in peripheral blood before and after IVIG treatment in responders and non-responders in peripheral blood. **(A)** The number of dominant BcR clones and **(B)** the impact of all dominant BcR clones before IVIG treatment. **(C)** the impact of all dominant BcR clones after IVIG treatment. *P < 0.05. Box plots show the median and 25th and 75th interquartile, error bars show the range. Single dots in grey represent values for each patient

We subsequently analysed whether BcR repertoire characteristics were associated with treatment response. The number of dominant clones before IVIG treatment was not different between responders and non-responders (response defined as TIS score ≥40) (Fig. 3A). Of interest, the impact of all dominant clones (cumulative frequency) before IVIG treatment was significantly higher in responders compared with non-responders (*P* < 0.05; Fig. 3B). Among the six patients without dominant clones at baseline, three were responders (Supplementary Table S2, available at *Rheumatology* online). Additionally, in comparison to the other responders, we did not see any differences in the two responders who had the highest impact of dominant clones at baseline in terms of clinical characteristics (Supplementary Table S2, available at *Rheumatology* online) or BcR features such as CDR3 length, CDR3 charge and V and J gene usage (data not shown). The two patients with the highest impact of all dominant clones before treatment (Fig. 3B) are two patients with immune-mediated necrotizing myopathy (IMNM), one with HMGCR antibodies and the other patient was seronegative.

In muscle biopsies, the impact of dominant clones did not show a correlation with response. Nine weeks after treatment, we did not observe this difference as the impact of all dominant BcR clones was comparable between responders and non-responders (Fig. 3C). In conclusion, based on a limited set of patients (*n* = 19), we observed a significantly higher impact of dominant BcR clones in blood before start of therapy in responders compared with non-responders to IVIG therapy.

### Correlation of BCR clonality with disease activity and response

**Figure 4.**
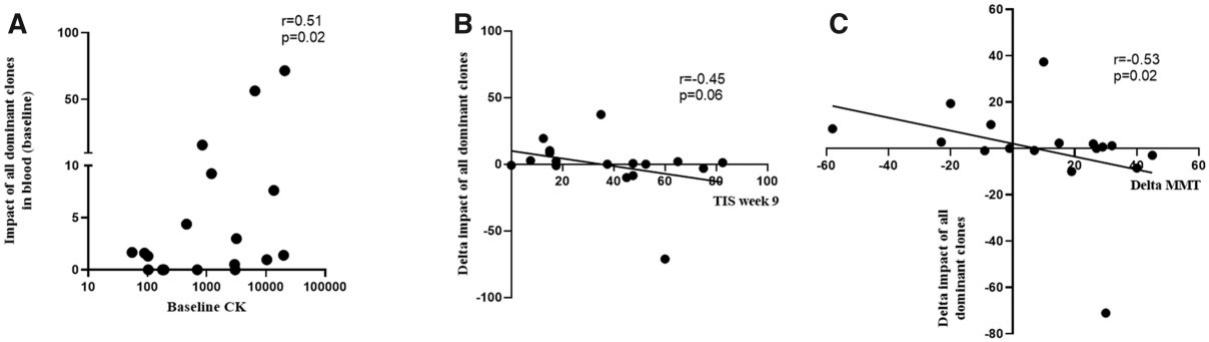
Correlation of BcR repertoire characteristics before and after treatment with markers of disease activity and therapy response. **(A)** The impact of all dominant BcR clones in blood at baseline *vs* CK levels at baseline. Correlation of the difference in impact of all dominant BcR clones in blood (week 9 minus week 0) with **(B)** the total improvement score (TIS) and **(C)** the improvement in manual muscle testing (MMT). *P* and r values are shown for each curve

Next, we assessed whether BcR repertoire features correlated with various markers of disease activity or therapy response. At baseline the impact of the dominant clones in blood correlated with increased CK in blood [Fig. 4A; r = 0.51; *P* = 0.02, 95% CI (0.08, 0.78)]. This was also reflected in a non-significant trend to association of CK with the number of dominant clones in peripheral blood (Supplementary Fig. S4B, available at *Rheumatology* online). Here, the number of dominant clones but not the impact of these clones also showed a significant inverse association with baseline MMT [Supplementary Fig. 4A, available at *Rheumatology* online; r = –0.46, *P* = 0.04, 95% CI (–0.76, 0.008)]. In muscle tissues, we did observe a non-significant [r = 0.36; *P* = 0.08, 95% CI (–0.16, 0.70)] trend in correlation between baseline MMT and impact of dominant clones (Supplementary Fig. S5, available at *Rheumatology* online). There was no significant correlation between the number and impact of dominant clones in muscle tissues at baseline with MMT and CK (Supplementary Fig. S4E and F, available at *Rheumatology* online).

After IVIG treatment, in blood a decrease in the *impact* of dominant clones correlated with an increase in MMT [Fig. 4C; r = 0.53; *P* = 0.02, 95% CI (–0.8, –0.12)]. and non-significantly with a better TIS response [Fig. 4B; r = 0.45; *P* = 0. 06, 95% CI (–0.77, 0.05)]. The *number* of dominant clones in blood did not correlate with CK, MMT or TIS (Supplementary Fig. 4C and D, available at *Rheumatology* online). In patients who shared dominant clones between muscle tissues and blood (*n* = 3), no significant correlations between the number and impact of shared dominant clones (blood-muscle) and TIS, MMT or CK were demonstrated (data not shown). There was no significant correlation between the number and impact of dominant clones in muscle tissues at baseline clinical response at follow-up (data not shown).

In conclusion, the impact of dominant BcR clones in blood correlated with higher CK levels at baseline. After IVIG, a decrease in the impact of dominant clones in blood correlated with higher total improvement score (TIS) as well as better muscle strength (MMT).

## Discussion

In this prospective exploratory study, we identified expanded BcR clones in muscle tissues of IIM patients prior to treatment, which confirms a report on treatment-naive dermatomyositis and polymyositis patients [18]. In addition, our study shows a possible relation between BcR clonality in peripheral blood and response to treatment, which was not described in the previous report because it focused on muscle tissue. We also observed expanded BcR clones in peripheral blood of IIM patients, and some expanded BcR clones present in muscle tissues could be found in peripheral blood. To the best of our knowledge, we report for the first time the presence of B cells carrying the same BcR signatures in muscle tissue and peripheral blood of treatable myositis patients. This finding suggests that B cells have the ability to migrate between peripheral blood and muscle tissues of myositis patients, as observed before with T cells in inclusion body myositis [36]. It is worth noting that some dominant BcR clones were muscle restricted as they were not detected in peripheral blood. This raises an intriguing question as to whether these muscle-restricted BcR clones express high affinity homing receptors which enable their firm attachment to muscle tissues, or whether they even proliferate and differentiate locally. Future work addressing this question may lead to a better understanding of the phenotype of B cells in muscle tissues of myositis patients. Additionally, whether tissue-restricted or overlapping BcR clones are disease-associated and whether they produce myositis-related antibodies warrants further investigation. Because the pathogenesis of myositis is different for each antibody, we investigated whether different myositis subtypes would show different BcR clonality patterns or repertoire features. We did not find any significant differences between the different myositis subtypes in terms of BcR clonality or BcR repertoire features. A possible explanation could be the limited number of patients in our pilot study.

The analysis of the BcR repertoire in muscle tissue revealed the presence of highly mutated BcR clones when compared with BcR clones retrieved from peripheral blood, indicating the presence of matured cells of the B-cell lineage in muscle tissue. This is in line with previous literature [18] and expected because organized lymphoid structures are present in tissues of myositis patients [37, 38], which contain matured (and thus mutated) plasma and memory B-cell subsets that may contribute to autoantibody production.

We also described the preferential usage of certain V-J gene rearrangements in the BcR clones present in both muscle tissues and peripheral blood. Preferential V gene usage by B-cell clones has been described in various autoimmune diseases such as RA, SLE as well as in myositis [39–41]. The preferential usage of certain V genes was described before in a cohort of 12 myositis patients [42]. Several reasons including antigen structure as well as antigen availability have been put forward as plausible reasons for this observed preferential usage of V genes in autoimmune conditions [43]. It is therefore tempting to speculate that this observation in our study is due to a selective response to specific antigens.

Another finding from our study was the presence of completely different dominant BcR clones before and after IVIG treatment. Similar findings have been observed in Guillain Barré syndrome patients treated with IVIG [44]. Their results suggest that IVIG can induce a *de novo* B-cell response that can be observed after treatment. These results are contrary to another study that investigated the effects of IVIG on the B-cell repertoire in patients undergoing desensitization therapy before transplantation and reported no major changes in the BcR repertoire after IVIG treatment [45]. Several reasons including dose as well as time of sampling could account for these differences. However, the authors did not describe whether BcR clones present before IVIG treatment were present after IVIG treatment.

Finally, an intriguing question is whether the B-cell repertoire both before and after IVIG treatment is associated with clinical benefits in myositis patients. In our study, we found that patients who had a high impact (cumulative frequency) of dominant BcR clones before treatment responded better to IVIG treatment. Of note, the impact of dominant BcR clones in blood also correlated with higher CK levels at baseline, and a decrease in the impact of dominant clones in blood correlated with higher total improvement score (TIS). This might suggest that patients exhibiting a dominant B-cell response in the pre-treatment repertoire do respond better on IVIG. However, our data should be interpreted with care as the number of patients included in our study is limited. A study in Guillain Barré syndrome patients reported an association between IVIG-induced plasmablasts after treatment and clinical recovery [44]. We did not find such a correlation between IVIG-induced dominant B-cell clones after treatment and clinical benefit. Further research with appropriate controls is needed to confirm these early observations.

Our study has some limitations. Firstly, the relatively low number of patients did not allow subgroup analysis for different myositis subtypes. Therefore, our observations need additional confirmation in larger patient cohorts that allow subgroup analysis. Secondly, because we did not make use of conventional flow cytometry in our study, our analysis yields little information on the phenotype of these dominant BcR clones. Thirdly, the lack of a control group precludes us from making firm conclusions on whether the effects on the BcR repertoire seen in this study are totally due to IVIG or simply due to the passage of time. Finally, our BcR repertoire technique is not able to determine the specificity and pathogenicity of these dominant BcR clones present both before and after IVIG treatment as well as in muscle tissues. For example, if the clones are related to the formation of myositis-related antibodies or are directly involved in the disease pathogenesis. As a result, extensive approaches such as single-cell sequencing coupled with recombinant antibody expressions as well as cell stimulation assays are needed to fully unravel the specificity and pathogenicity of these dominant clones.

## Conclusions

In conclusion, we have shown that BcR clones present in muscle tissues can be retrieved in peripheral blood of IIM patients. Although not associated with treatment response, in peripheral blood, new patient-specific dominant BcR clones were formed during IVIG treatment and, pre-treatment dominant BcR clones disappeared after treatment. Finally, the high impact of dominant B-cell clones before treatment is associated with a better clinical response to IVIG, suggesting that response to IVIG might depend on the pre-treatment BcR repertoire. Future work tailored towards a better understanding of the pre-treatment BcR repertoire in myositis patients might result in the identification of biomarkers that could predict eventual response to IVIG treatment.

## Supplementary Material

keac602_Supplementary_Data

## Data Availability

The dataset generated and presented in this study can be retrieved online. The repository and accession number can be found in the following link: https://www.ncbi.nlm.nih.gov/, PRJNA814462.
