# Supplementary material for: B-cell receptor profiling before and after IVIG monotherapy in newly diagnosed idiopathic inflammatory myopathies
Source: Rheumatology (Oxford). 2022 Nov 2;62(7):2585–93. doi: 10.1093/rheumatology/keac602 (PMC10321087; doi:10.1093/rheumatology/keac602)
Supplement: keac602_Supplementary_Data [file keac602_supplementary_data.zip › keac602_Supplementary_Data/rhe-22-0791-File002.docx]

*Supplementary materials and methods*

*Clinical assessment*

The six core set measures of the IMACS score used for clinical examination included: : the patient global activity assessment (PGA), Physician global activity assessment (PhGA), Health Assessment Questionnaire (HAQ), extra muscular disease activity based on the Myositis Disease Activity Assessment Tool (MDAAT), muscle enzymes and muscle strength testing according to manual muscle testing (MMT 13) in which a score of 10 reflects maximal strength in the individual muscle, and the total score per patient ranged from 0-260 (1). At follow up, a change score (follow up–baseline) was calculated for MMT and CK. Clinical improvement after treatment was measured by the total improvement score (TIS) ranging from 0-100, with higher scores reflecting more improvement (1), and a score ≥40 reflecting clinically significant response to IVIg treatment.

*RNA isolation and Next generation sequencing of the BcR repertoire*

Following RNA isolation from peripheral blood, muscle biopsies (stored in -80°C) were homogenized by spinning twice with 900µl of RLT buffer (1% B-mercaptoethanol) and ceramic beats in a MagNA Lyser (Roche) at 6500rpm for 30 seconds with cooling on ice for 1 minute in-between. The cell lysate was incubated with 60µl of Proteinase K and 240µl RNase free water for 10 minutes at 55°C on a shaking heat block. RNA was extracted from the resulting cell lysate using the RNeasy Mini kit (Qiagen, Hilden, Germany), Complementary DNA of BcR heavy chain molecules was synthesized using a BcR heavy chain Joining reverse primer tagged with a 9 random nucleotide UMI and a consensus sequence, followed by Exonuclease I treatment (Thermo Fisher Scientific, Breda, The Netherlands) to remove left over primers. This was followed by a PCR with forward primers covering the BcRh Variable genes and a reverse primer binding to the consensus sequence previously introduced in the specific-cDNA and tagged with an 8 bp patient identifier (MID, Molecular Identifier). Obtained amplicons were purified using two rounds of AMPure XP beads clean-up (Beckman Coulter, Woerden, The Netherlands), quantified using Qubit dsDNA HS Assay Kit (Thermo Fisher Scientific), dual-indexed with i5 and i7 adapters (Nextera XT Index Kit v2) and sequenced using the Illumina Miseq Kit v3 2 x 300 bp technology according to the manufacturer’s manual (Illumina, San Diego, California, USA).

*Bioinformatics generation and analysis of BcR repertoires*

The generation of BcR repertoires from the RESEDA pipeline (REpertoire SEquencing Data Analysis, <https://bitbucket.org/barbera/reseda>) was carried out using the following steps: 1) pairwise assembly of the paired-end reads using PEAR (2), 2) identification of the 8 bp MID, 3) identification of the complement determining region 3 (CDR3), 4) alignment to the IMGT database (3) to obtain the Variable and Joining gene assignment, 5) removal of reads with low quality bases (Q score < 30) in the CDR3, 6) clustering of reads in clones based on 100% amino acidic CDR3 identity, 7) UMI-based correction of clonal frequencies and 8) contamination check between samples from different individuals. The final list of clones obtained from the RESEDA pipeline were analyzed with in-house developed R scripts using R version 4.1 (4) using R studio (5). The frequency of each clone was calculated as percentage of the total number of reads with unique UMIs obtained from sequencing of that sample. Clones with a frequency greater than or equal to 0.5% of the total repertoire were labelled dominant or highly expanded clones (HECs) (6, 7). The impact of a clone was calculated as its UMI-corrected frequency in the repertoire, and the impact of a group of clones as their cumulative frequency.

*Clustering related clones*

A dynamic threshold was determined that was used to cluster clones. For every sample a density function was calculated over the Hamming distance matrix. This resulted in a bimodal distribution. The minimum between the two peaks was taken as a threshold with a minimum value of 3 amino acids. Clones with a Hamming distance below the dynamic threshold were grouped together to form a cluster and therefore considered to be related to each other.

*Evaluation of diversity*

To evaluate the diversity between the repertoires, the Simpson’s index, Shannon entropy and the Gini index were used. While the Simpson’s index and Shannon entropy are able to measure the diversity in the BcR repertoires with a larger score indicating a higher diversity, the Gini index is able to measure the heterogeneity of different clones in a sample; it is bound between 0 and 1. A Gini index of 0 indicates that all the clones in the samples have the same frequency (i.e., all clones have equal number of clustered reads), whereas an index of 1 indicates that the reads in the sample are more clustered towards individual or specific clones.

Supplementary References

1. Aggarwal R, Rider LG, Ruperto N, Bayat N, Erman B, Feldman BM, et al. 2016 American College of Rheumatology/European League Against Rheumatism Criteria for Minimal, Moderate, and Major Clinical Response in Adult Dermatomyositis and Polymyositis: An International Myositis Assessment and Clinical Studies Group/Paediatric Rheumatology International Trials Organisation Collaborative Initiative. Arthritis Rheumatol. 2017;69(5):898-910.

2. Zhang J, Kobert K, Flouri T, Stamatakis A. PEAR: a fast and accurate Illumina Paired-End reAd mergeR. Bioinformatics. 2014;30(5):614-20.

3. Giudicelli V, Chaume D, Lefranc MP. IMGT/GENE-DB: a comprehensive database for human and mouse immunoglobulin and T cell receptor genes. Nucleic Acids Res. 2005;33(Database issue):D256-61.

4. R Core Team. A language and environment for statistical computing. R Foundation for Statistical Computing. Vienna, Austria2021.

5. RStudio Team. RStudio: Integrated Development for R. RStudio, PBC. Boston, MA2020.

6. Pollastro S, Klarenbeek PL, Doorenspleet ME, van Schaik BDC, Esveldt REE, Thurlings RM, et al. Non-response to rituximab therapy in rheumatoid arthritis is associated with incomplete disruption of the B cell receptor repertoire. Ann Rheum Dis. 2019;78(10):1339-45.

7. Klarenbeek PL, Tak PP, van Schaik BDC, Zwinderman AH, Jakobs ME, Zhang Z, et al. Human T-cell memory consists mainly of unexpanded clones. Immunology Letters. 2010;133(1):42-8.
