# Supplementary material for: B-cell receptor profiling before and after IVIG monotherapy in newly diagnosed idiopathic inflammatory myopathies
Source: Rheumatology (Oxford). 2022 Nov 2;62(7):2585–93. doi: 10.1093/rheumatology/keac602 (PMC10321087; doi:10.1093/rheumatology/keac602)
Supplement: keac602_Supplementary_Data [file keac602_supplementary_data.zip › keac602_Supplementary_Data/rhe-22-0791-File003.docx]

# Supplementary Tables

**Supplementary Table S1**: CDR3 amino acid sequences of BcR clones dominant in both muscle tissues and peripheral blood prior to IVIg treatment.

| CDR3 AA sequence | Patient | Clone size in muscle tissue (%) | Clone size in blood (%) | IGHV gene | JH gene |
| --- | --- | --- | --- | --- | --- |
| CVKDLGDDYGANPRRFDYWGQGTLVT | PT1 | 2.72 | 3.9 | 3-64 | 4 |
| CARILARSVFPFDYWGQGTLVT | PT1 | 24.6 | 3.5 | 4-31 | 4 |
| CARAPPFRYVLRFLDDPHYFDYWGQGTLVT | PT9 | 6.8 | 35.6 | 1-2 | 4 |
| CAGSEYSSSWFPFRGPRPPLTNWGQGTLVT | PT9 | 0.5 | 12.9 | 3-3 | 4 |
| CTRASSGWYVGNNWFDPWGQGTLVT | PT12 | 1.2 | 1.5 | 3-49 | 5 |

## Supplementary Table S2: BcR clonality at baseline for the different patient subgroups

| **Characteristic** | Myositis specific  antibody (MSA) (n=11) | Myositis associated  antibody (MAA) (n=5) | Seronegative (n=3) |
| --- | --- | --- | --- |
| Number of clones blood (baseline) median (IQR) | 4402 (3462-5071) | 4643 (3140-5524) | 5111 (1699-5690) |
| Impact of dominant clones blood (baseline) median (IQR) | 1.4 (0-7.63) | 1.7 (0.5-10.1) | 0.0 (0-56.4) |
| Number of clones muscle (baseline) median (IQR) | 516 (436-936) | 442 (343.5-616.0) | 431 (374-495) |
| Impact of dominant clones muscle (baseline) median (IQR) | 58.6 (41.6-66.9) | 73.8 (63.2-79.5) | 69.2 (64.0-70.7) |

## Supplementary Table S3; Clinical characteristics of responders and non-responders

| **Characteristic** | Responders (n=8) | Non responders (n=11) |
| --- | --- | --- |
| Age at onset in years, median (IQR) | 62 (39 – 70) | 56 (37 – 67) |
| Months between start of symptoms until diagnosis, median (IQR) | 4 (1.5 – 7) | 5 (4 – 6) |
| Gender, females, n (%) | 5 (63) | 7 (64) |
| Connective tissue disorder, n (%) | 1 (13) | 2 (18) |
| Cancer, n (%) | 1 (13) | 2 (18) |
| Serum CK, times normal value of CK (mean, SD)¤ | 22 (31) | 20 (32) |
| Responders, TIS ≥40 at 9 weeks, n (%) | 8 (42) | 11 (58) |
| Number of dominant clones blood (baseline) median (IQR) | 3 (0-4) | 1 (0-3) |
| Impact of dominant clones blood (baseline) median (IQR) | 9.2 (0-46.43) | 1.2 (0.0-1.7) |
| Number of dominant clones muscle (baseline) median (IQR) | 67.2 (60.1-73.48) | 65.1 (45.5-78.3) |
| Impact of dominant clones muscle (baseline) median (IQR) | 69.9 (59.9-73.0) | 61.4 (45.5-76.4) |

## IQR interquartile range; CK creatine kinase; ¤ according to a CK reference value <217U/L. Supplementary Figures

## Supplementary Figure S1


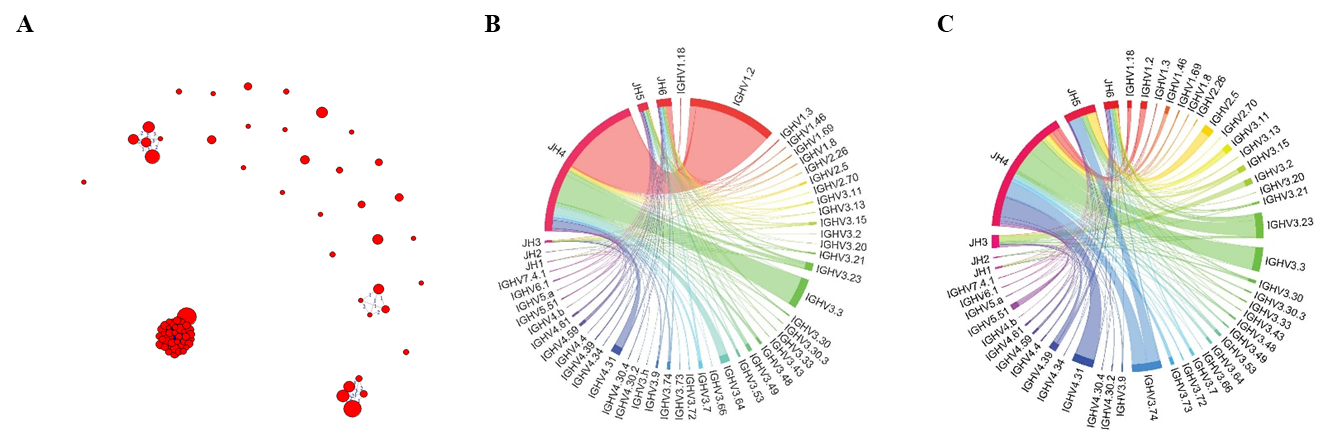


**Supplementary Figure S1:** **Characteristics of BcR clones present in both muscle tissues and peripheral blood prior to IVIg treatment**. **(A)** RepresentativeCDR3 network analysis of top 100 most expanded clones present in both peripheral blood and muscle tissues prior to IVIg treatment. Data is shown for one patient. V and J gene usage in **(B)** peripheral blood and **(C)** muscle tissues for BcR clones present in both peripheral blood and muscle tissues prior to IVIg treatment. Data is shown for all 19 patients.

## Supplementary Figure S2

**Supplementary Figure S2:** **BcR repertoire characteristics in peripheral blood before and after IVIg treatment**. For each patient panel **(A&D)** shows the number (upper row) and impact of dominant BcR clones (lower row) in patients with myositis specific antibodies (MSA). **(B&E)** showsthe number and impact of all dominant BcR clones in patients with myositis associated antibodies (MAA) and, **(C&F)** shows the number and impact of all dominant BcR clones in patients who were seronegative.

## Supplementary Figure S3

**A**

**B**


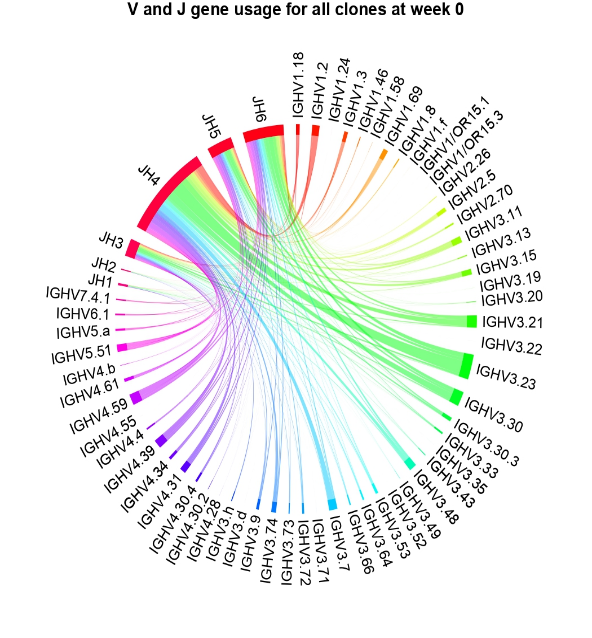

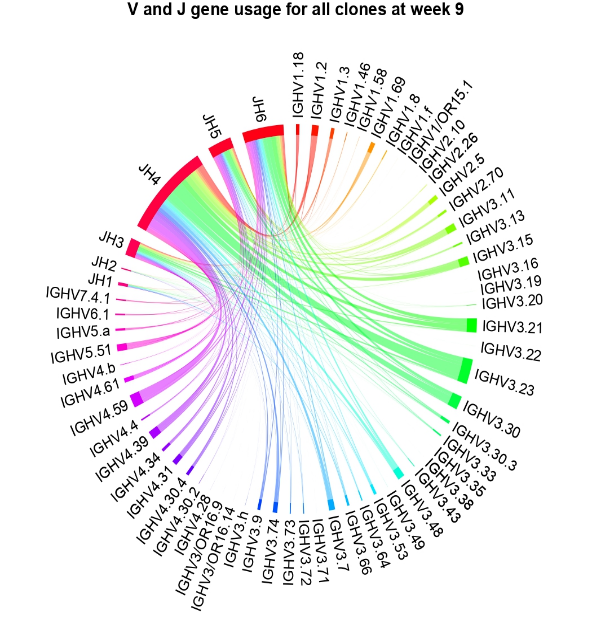


**C**

**D**

**E**

**Supplementary Figure S3:** **Characteristics of the BcR repertoire in peripheral blood prior to and 9 weeks after IVIg treatment**. Overall **(A)** CDR3 length and **(B)** CDR3 charge of BcR clones detected in peripheral blood before and 9 weeks after IVIg treatment. Data is shown for all 19 patients. **(C)** V and J gene usage for BcR clones present before (left panel) and 9 weeks (right panel) after IVIg treatment. Data is shown for all 19 patients. **(D)** CDR3 overlap plot is shown for 1 patient in which the same dominant BcR clone was present before and 9 weeks after IVIg treatment. Each symbol represents a unique BcR clone, and its frequency in the analyzed repertoire is depicted on the x-axis (blood pre-IVIg treatment) and y-axis (blood post-IVIg treatment) as percentage of total reads. The dotted red lines on each axis indicate the 0.5% cut-off for dominant BcR clones. Dominant BcR clone present before and after IVIg treatment is colored in green. **(E)** Representative CDR3 overlap plot for two 2 different patients 9 weeks after IVIg treatment. Colored clones in each patient are newly formed dominant BcR clones. (F-G) Simpson’s index, Shannon entropy and Gini index to evaluate the diversity of the BcR repertoires pre and post IvIg treatment.

**Supplementary Figure S4**

**Supplementary Figure S4:
Correlation of BcR repertoire characteristics before and after treatment with markers of disease activity and therapy response**. The number of dominant BcR clones in blood at baseline versus **(A)** baseline MMT and **(B)** baseline CK. The number of dominant BcR clones in blood at week 9 versus **(C)** MMT at week 9, **(D)** CK at week 9. The number of dominant BcR clones in muscle tissues at baseline versus **(E)** baseline MMT and **(F)** baseline CK. MMT; manual muscle testing; CK; creatine kinase p and r values are shown for each curve.

## Supplementary Figure S5

**Supplementary Figure S5**: Correlation of the impact of dominant BcR clones in muscle tissues at baseline and the baseline MMT (muscle strength). MMT; manual muscle testing.
